# Porphyrin-based polyimide 2D porous organic polymers: band engineering for bifunctional electrocatalytic OER and HER

**DOI:** 10.1039/d5ma00957j

**Published:** 2025-08-29

**Authors:** Deepak Bansal, Amr A. Nada, Samrat Ghosh, Indresh Kumar Pandey, Nicolas D. Boscher

**Affiliations:** a Materials Research and Technology Department, Luxembourg Institute of Science and Technology 28 Avenue des Hautes-Fourneaux Esch-Sur-Alzette Luxembourg Deepakbans@gmail.com amr.nada@list.lu; b Inorganic and Physical Chemistry Laboratory, Council of Scientific and Industrial Research (CSIR), Central Leather Research Institute (CLRI) Chennai 600020 India; c Department of Chemistry, D.D.U Gorakhpur University Gorakhpur Uttar Pradesh India

## Abstract

The development of bifunctional catalysts for overall water splitting is a rapidly advancing area of research. In this study, we report the synthesis of novel porphyrin-based polyimide polymers for electrocatalytic oxygen (OER) and hydrogen evolution reactions (HER). By assembling aminophenyl porphyrins with naphthalenetetracarboxylic dianhydride (NTCDA) linkers, donor–acceptor (D–A) architectures that enable extended π-electron delocalization and tailored frontier molecular orbitals conducive to water splitting were formed. FTIR, XPS and XRD analyses confirmed successful imidization between aminophenyl porphyrins (TAPP, NiTAPP, and CuTAPP) and NTCDA, resulting in π–π stacked two-dimensional polymer networks POP-1, POP-2, and POP-3, respectively. Band structure studies *via* XPS revealed that both imidization and metal incorporation significantly influence the electronic properties and enable the fine-tuning of the HOMO–LUMO levels for enhanced electrocatalytic activity. Electrochemical evaluations demonstrated the bifunctional nature of the polymers, with the Ni(ii)-based polymer, *i.e.*POP-2, showing the best performance. POP-2 exhibited a lower OER onset potential (1.45 V *vs.* RHE) and smaller Tafel slope (167 mV dec^−1^) compared to its metal-free (POP-1) and Cu(ii)-based (POP-3) analogues. Similarly, in the HER, POP-2 displayed a reduced onset potential (*ca.* 159 mV) and a lower Tafel slope (*ca.* 82 mV dec^−1^), attributed to the favorable redox behavior of Ni, optimal hydrogen binding energy, and enhanced charge delocalization. Electrochemical impedance spectroscopy confirmed its superior conductivity, while Mott–Schottky and XPS analyses revealed beneficial band alignment and increased charge carrier density. Long-term stability tests further validated the durability of POP-2. This work highlights the potential of metal-coordinated conjugated polymers as efficient and robust heterogeneous bifunctional electrocatalysts for overall water splitting.

## Abstract 2

Porphyrin-based polyimide polymers as bifunctional electrocatalysts for oxygen (OER) and hydrogen evolution reactions (HER).

## Graphical abstract

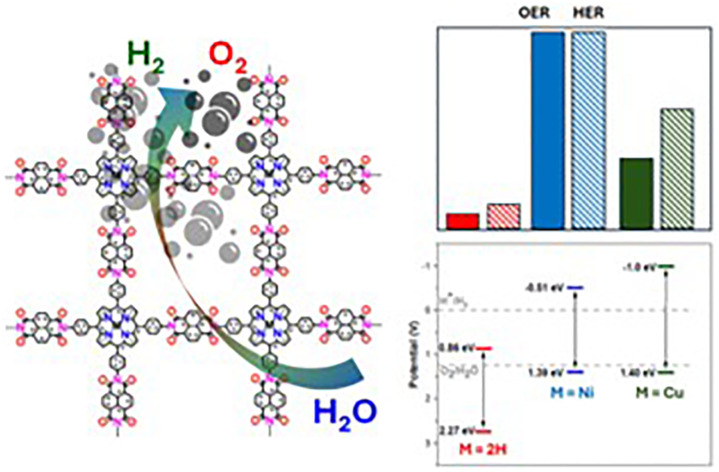

## Introduction

The development of efficient and stable electrocatalysts for energy conversion reactions, such as the oxygen evolution (OER) and hydrogen evolution (HER) reaction, is a major focus in the pursuit of renewable energy technologies including water splitting and fuel cells.^1–5^ The overall water splitting reaction proceeds *via* two half-cell reactions: the cathodic reduction of protons (2H^+^ + 2e^−^ → H_2_) and the anodic oxidation of water (2H_2_O → O_2_ + 4H^+^ + 4e^−^).^1,6–11^ Among these, the OER is generally considered the more kinetically sluggish due to the involvement of a complex four-electron, four-proton transfer process, inducing a higher overpotential compared to the HER.^12–15^ While noble metal-based catalysts such as Pt/C, IrO_2_, and RuO_2_ demonstrate excellent activity for the HER or OER, their high cost, limited stability, and single-function nature restrict their widespread application.^12,16–21^ Consequently, research has increasingly focused on earth-abundant alternatives, including transition metal-based catalysts and heteroatom-doped carbon materials.^22–26^ However, these systems often suffer from either poor durability or difficulty in controlling the precise doping level and spatial distribution of the active sites, limiting their catalytic performance and mechanistic understanding. A promising strategy is the incorporation of transition metal ions (*e.g.*, Co^2+^, Ni^2+^, Fe^2+^) into organic coordination environments, enabling the design of multifunctional electrocatalysts with tunable activity toward the HER or OER.^1,27–30^ Within this context, porous organic materials such as metal–organic frameworks (MOFs) and covalent organic frameworks (COFs) have attracted significant attention due to their high surface area, structural tunability, and potential applications in energy storage, sensing, and catalysis.^31–39^ In particular, porphyrin-based porous organic polymers (POPs) are of great interest because of their highly conjugated π-systems and rich redox chemistry.^40–44^ Functionalizing the porphyrin core with different metal centres or modifying its meso- and β-positions enables the fine tuning of its electronic and catalytic properties.^1^ Furthermore, strong π–π interactions between porphyrin units and their assembly as extended conjugated assemblies both facilitate the formation of two-dimensional architectures with high conductivity and accessibility of active sites.^6,45^

A key factor governing catalytic performance in such systems is the alignment of frontier molecular orbitals (FMOs), specifically the valence and conduction band positions, with the targeted catalytic reactions.^48–50^ For efficient overall water splitting, the valence band must lie below the oxidation potential of water (O_2_/H_2_O), and the conduction band above the reduction potential of protons (H^+^/H_2_).^46–51^ Designing porphyrin-based POPs with precisely aligned FMOs for both the HER and OER remains a significant challenge. However, this can potentially be addressed through rational molecular engineering of donor–acceptor (D–A) architectures.^52–56^ In this regard, naphthalene diimide (NDI) is a particularly attractive acceptor unit due to its electron-deficient nature, excellent redox stability, high electron affinity, and good charge mobility.^57^ When incorporated into conjugated frameworks alongside electron-rich porphyrins, NDI can enhance charge separation, transport, and the overall catalytic performance of the resulting material.^58–60^ The unique properties of these building blocks are low lying FMOs, which are expected to tune the HOMO–LUMO position of the resulting polymer(s) and enhance their catalytic performance for both oxygen evolution and proton reduction reactions, offering a potential pathway for sustainable energy conversion and storage.

The most used synthetic techniques are poly-condensation reactions involving either boronic acids^3^ or amine and aldehyde groups to prepare C–C^61,62^ coupled and C=N^63,64^ linked porphyrin-based POPs. The difficulties in large scale synthesis of C–C coupled building blocks due to harsh reaction conditions and low hydrothermal stability of –C=N– linkage are serious limitations to their practical application. Recently, novel polyimide (PI)-based porphyrin POPs have been synthesized through the immidization reaction for drug delivery,^65^ Suzuki–Miyaura coupling reactions,^60^ sensing^66^ and Zn^2+^ ion batteries.^67^ PI-based POPs as compared to other COFs, show excellent thermal stability, well crystalline, large pore sizes, and high surface area. Surprisingly, despite their enormous potential, the investigation of the synthesis of porphyrin-based PI-POPs and their electrocatalytic properties are largely unexplored.^66^

In this work, we report the facile synthesis of three donor–acceptor POPs TAPP-NDI (POP-1), NiTAPP-NDI (POP-2), and CuTAPP-NDI (POP-3) by combining tetra(aminophenyl)porphyrins (TAPP, Ni^II^TAPP or Cu^II^TAPP) as an electron-donating unit and naphthalenetetracarboxylic dianhydride (NTCDA) as an electron-accepting unit *via* a polyimide linkage. Structural characterization using FTIR, XPS, and PXRD confirmed the successful formation of the imidized frameworks and microcrystalline features. Electrochemical evaluation revealed that the Ni(ii)-chelating POP-2 exhibits significantly enhanced bifunctional catalytic performance for both the HER and OER compared to the metal-free (POP-1) and Cu(ii)-chelating (POP-3) analogues. This superior activity is attributed to the optimal alignment of FMOs enabled by the donor–acceptor architecture and the catalytic versatility of the Ni centre. Our findings highlight the promise of porphyrin–NTCDA polyimide POPs as efficient and robust catalysts for overall water splitting and energy conversion applications.

## Results and discussion

### Synthesis and characterization of the 2D porous organic polymers

**Fig. 1:**
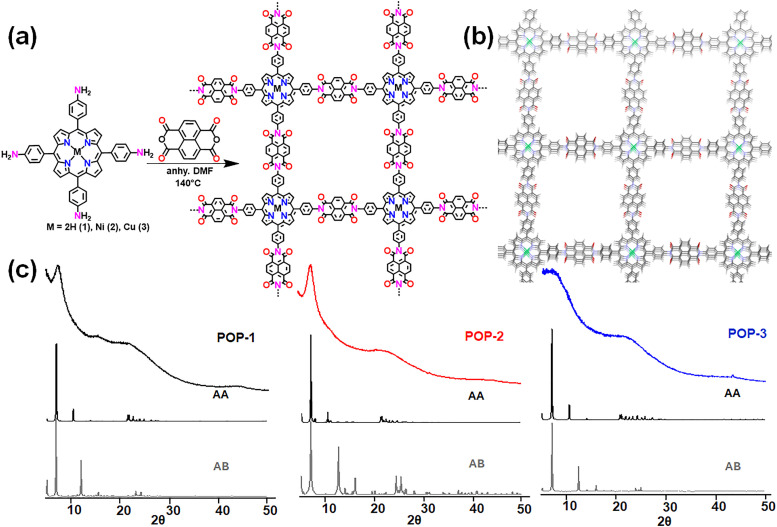
Synthesis of the porous organic polymers. (a) Schematic representation of the synthesis of POPs 1–3. (b) representative eclipsed stacked structure for the POP. (c) PXRD data for the synthesised POPs along with their simulated pattern in eclipsed (AA) and staggered (AB) form.

The 2D porous organic polymers were synthesized *via* a condensation reaction between 1,4,5,8-naphthalenetetracarboxylic dianhydride (NTCDA) and three different porphyrin-based monomers: 5,10,15,20-tetrakis(4-aminophenyl)porphyrin (TAPP), Ni(ii)-5,10,15,20-tetrakis(4-aminophenyl)porphyrin (NiTAPP), and Cu(ii)-5,10,15,20-tetrakis(4-aminophenyl)porphyrin (CuTAPP) (Fig. 1a). The reactions were carried out in dimethylformamide (DMF) at 140 °C for 48 hours under dark conditions. This process yielded insoluble precipitates of the corresponding polyimide-based porous organic polymers: NDI-TAPP (POP-1, 66.0%), NDI-NiTAPP (POP-2, 78.0%), and NDI-CuTAPP (POP-3, 75.0%), respectively. The high yields and insolubility of the products are consistent with successful polymerization *via* imidization. The FTIR spectra of the as-synthesized polymers confirm the successful formation of imide linkages through the appearance of characteristic asymmetric and symmetric C=O stretching vibrations of the imide ring at approximately 1780 and 1714 cm^−1^, respectively (Fig. S1–S3). Additionally, a strong absorption band at *ca.* 1340 cm^−1^, attributed to the C–N–C stretching within the imide structure, is observed. The complete disappearance of the –NH_2_ stretching band near 3240 cm^−1^ further supports the successful imidization of NTCDA with the aminophenyl-substituted porphyrins.^65^ To further confirm the chemical structure and investigate the local environment of the carbon atoms, solid-state ^13^C NMR spectroscopy was performed for the non-metalated polymer (POP-1) and the Ni(ii)-chelating polymer (POP-2), taking advantage of the diamagnetic nature of Ni(ii) (Fig. S4). Both polymers exhibit similar spectra, displaying multiple resonances in the range of 100–180 ppm, which are typical for aromatic and carbonyl carbons. A resonance around 160 ppm corresponds to the carbonyl carbons of the imide units. Additional signals at 116.8, 126.3, and 133.9 ppm are assigned to aromatic carbons from the phenyl and porphyrin units.^66^ Weak sideband signals observed at approximately 30 ppm and 230 ppm are attributed to spinning side bands, a characteristic of magic-angle spinning (MAS) in solid-state NMR and are commonly associated with anisotropic environments such as carbonyl groups in the naphthalenediimide units.

**Fig. 2:**
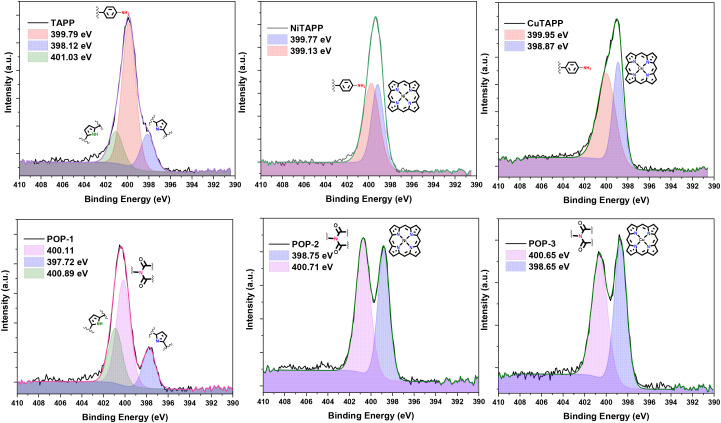
Comparative analysis of the change in N 1s core level for the porphyrin monomers (top) to their respective porous organic polymers (below).

The XPS analysis of the N 1s and O 1s regions provided crucial insights into the formation of polymeric structures.^68^ The N 1s XPS spectra of the powder sample, pressed onto carbon tape, exhibited core features within a binding energy (BE) range of 397–401 eV (Fig. 2a). As anticipated, TAPP revealed three N 1s core components, corresponding to three electronically distinct nitrogen species: protonated pyrrole nitrogen (–NH_pyr_) at 401.03 eV, unprotonated pyrrole nitrogen (–N_pyr_) at 398.12 eV, and amine nitrogen (–NH_2_) at 399.79 eV. In contrast, the presence of coordinated Ni(ii) and Cu(ii) in NiTAPP and CuTAPP, respectively, resulted in two closely positioned signals attributed to –N_pyr_ at 399.13 eV and 398.87 eV, and –NH_2_ at 399.77 eV and 399.95 eV, respectively. Notably, the condensation of the amine group with NTCDA to form an imide linkage was evidenced by the appearance of an N 1s core signal at 400.1 eV in POP-1 and 400.7 eV in POP-2 and POP-3. The approximately 1 eV shift in the N 1s BE value of –NH_2_ suggests its linkage to the electron-deficient NTCDA moiety. On the other hand, for the O 1s region, the XPS spectra of NTCDA displayed two distinct signals, corresponding to four carbonyl (–C=O) groups at 532.5 eV and two anhydride oxygen atoms at 529.1 eV (Fig. S5). A signal close to *ca.* 532.0 eV was also observed in all the samples, likely originating from oxygen on silicon wafer in the form of SiO_*x*_ or residual solvent molecules. Interestingly, the absence of an anhydride oxygen signal in POPs 1–3 indicates complete condensation between amine and NTCDA. Additionally, the –C=O signals in the porous polymers shifted to a lower BE by approximately 0.5 eV, which can be attributed to their linkage with the electron-rich porphyrin framework.

The crystallinity of the as-synthesised POPs was characterized by powder X-ray diffraction (PXRD) measurements. Interestingly, all the POPs exhibit broad diffraction signals indicating moderate to low crystalline nature of the POPs (Fig. 1c). As shown in the PXRD patterns, the most intense peak is observed at 7.35° for POP-1, 7.59° for POP-2 and 6.82° for POP-3, followed by broad features between 20 and 23°. Judging from the full width at half maximum (FWHM) of the first reflection at *ca.* 7°, POP-1 and POP-2 exhibit greater crystallinity than POP-3. While the feature corresponding to *ca.* 7° reflects the formation of two-dimensional (2D) crystalline frameworks, whereas the signal at *ca.* 22° indicates the pi-stacking between the 2D layers. In order to confirm the 2D framework formation, structural simulations were performed by considering both eclipsed (AA) and staggered (AB) arrangements. The experimental PXRD of the POPs closely matches with the simulated diffraction pattern of AA (Fig. 1b), in comparison to the AB arrangement.^69^ In addition, Field-emission scanning electron microscopy (FE-SEM) exhibits the presence of a highly dense microporous material, which is further reflected in the TEM images (Fig. S6). SEM–EDX elemental mapping confirms the homogeneous composition across the films. POP-2 and POP-3 display even Ni–C–N and Cu–C–N co-distributions, respectively (Fig. S7). No micron-scale metal aggregation is detected in the mapped areas. However, despite several attempts, HR-TEM did not enable the detection of an exploitable pattern, suggesting the formation of polymers with moderate crystallinity.

**Fig. 3:**
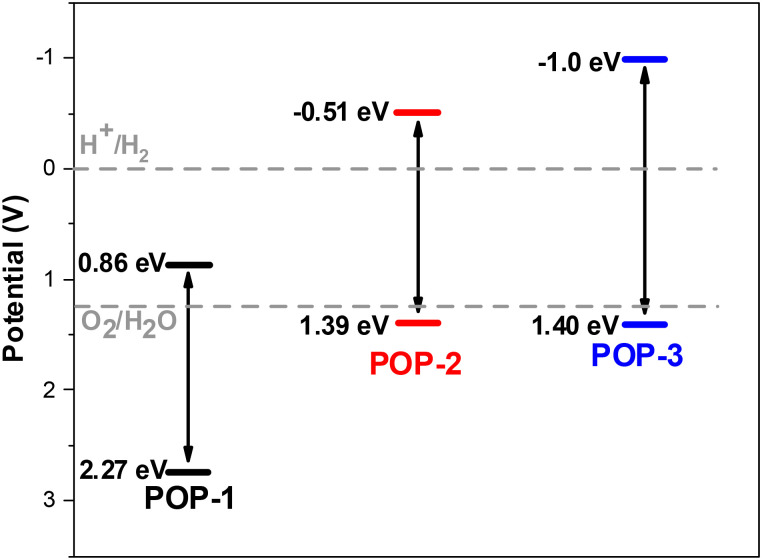
Schematic illustration of the band structures of the POPs 1–3.

The microporous characteristics and amorphous nature of the synthesized POPs were systematically investigated through nitrogen adsorption–desorption isotherms measured at 77 K. Prior to analysis, all samples were thoroughly degassed at 150 °C under vacuum for 2 hours to remove any residual solvents or moisture that might interfere with gas adsorption. The microporosity and amorphous nature of the POPs were further observed by Brunauer–Emmett–Teller (BET) and Barrett–Joyner–Halenda (BJH) Model analyses exhibiting low pore volume and surface area of 0.124 cc g^−1^ and 69.98 m^2^ g^−1^ in POP-1, 0.07 cc g^−1^ and 51.25 m^2^ g^−1^ in POP-2 and 0.69 cc g^−1^ and 218.35 m^2^ g^−1^ in POP-3 (Fig. S8). The relatively low values likely account for partial pore collapse during activation of the extended π–π stacking of the polyimide frameworks. Moreover, the unusually higher surface area of POP-3 is potentially attributed to (i) the smaller ionic radius of Cu^2+^ relative to Ni^2+^, which induces less distortion in the π–π stacking of the porphyrin units, and (ii) enhanced microporosity arising from improved packing efficiency. The imidization of the porphyrins with electron deficient NTCDA are observed in the absorption properties of the synthesised POPs. The diffused reflectance absorption of the POPs display a red shifted Soret band by *ca.* 17 nm in POP-1 and *ca.* 7 nm for POP-2 and POP-3 followed by broadening of the Q-bands, which most likely arises from the aggregation of 2D polymer sheets due to the strong π–π stacking property of both porphyrins and naphthalenediimides (Fig. S9). Moreover, the band gap values determined from the Tauc plot (Fig. S10) range from 1.90 eV for POP-1 and POP-2 to 2.40 eV for POP-3, which is significantly lower than their porphyrin counterparts TAPP (2.70 eV), NiTAPP (2.80 eV) and CuTAPP (2.83 eV), respectively. Interestingly, the HOMO–LUMO band positions derived from the combination of VBM measurement (Fig. S11) and Tauc plot display lowering in the HOMO level by 0.11 eV for POP-1 (2.27 eV), 0.21 eV for POP-2 (1.39 eV) and 0.45 eV for POP-3 (1.40 eV) whereas, the LUMO level decreases by 0.68 eV for POP-1 (0.86 eV), 1.11 eV for POP-2 (−0.51 eV) and 0.88 eV for POP-3 (−1.0 eV) with respect to their porphyrin counterparts TAPP, NiTAPP and CuTAPP emphasising the significant electronic effect of NTCDA on the electronic structure of the resulting porphyrin-based porous organic polymers (Fig. 3). Meanwhile, the introduction of metal ions in POP-2 and POP-3 resulted in the increase of the VBM by *ca.* 0.88 eV as compared to non-metalated POP-1. Gratifyingly, as anticipated, the combination of acceptor (NTCDA) with low-lying FMOs and electron donor (porphyrin) resulted in tuning the FMOs at an intermediate level of the individual band position of both the porphyrin and NTCDA, except for the HOMO level of POP-1, which remains at 0.86 eV, *i.e.* below the HOMO position of both TAPP and NTCDA (Fig. 3 and Fig. S12).

The structural insight towards the donor–acceptor (D–A) character of the synthesised POPs was analysed by performing DFT calculations with the B3LYP/LANL2DZ method using Gaussian 16. The results reveal that in POP-1 and POP-2, the FMOs are mainly localized on the porphyrin units and NDI linkers, consistent with a D–A framework (Fig. S13). Specifically, the HOMO of POP-2 is delocalized over the porphyrin unit, including the Ni(ii) center, while the LUMO is primarily localized on the NDI linker. In contrast, POP-3 exhibits a markedly different distribution (Fig. S14): both the HOMO and LUMO are localized on the N_4_–Cu core, with higher orbitals (HOMO+1/LUMO+1) showing a similar trend. Interestingly, while HOMO+2 continues to follow this delocalization pattern, LUMO+2 redistributes over the NDI linker. These findings highlight the distinctive influence of metal incorporation on orbital distribution, underscoring the synergistic roles of transition-metal centres and extended conjugation in governing catalytic activity.

### Electrocatalytic performance of POPs for water splitting applications

**Fig. 4:**
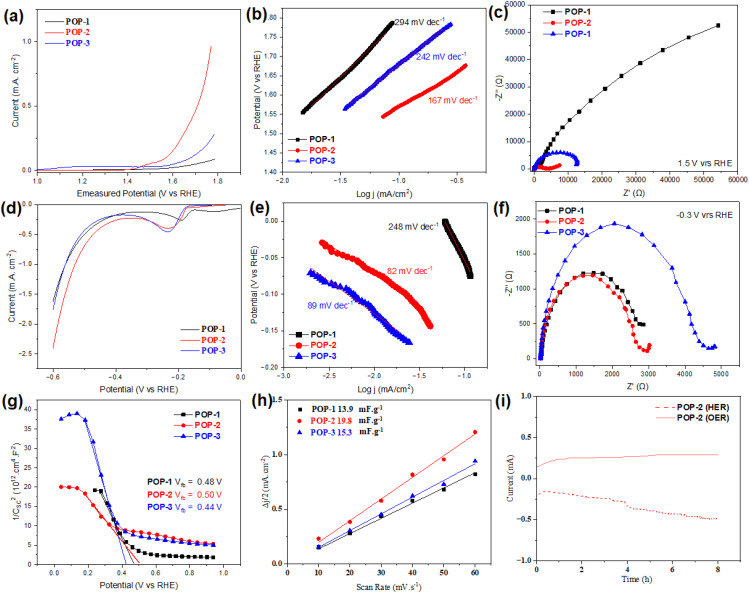
(a) and (d) LSV curves, (b) and (e) Tafel slopes, and (c) and (f) EIS measurements for the OER and HER, respectively, and (g) Mott–Schottky plot for POP-1, POP-2 and POP-3. (h) The *C*_dl_ values to evaluate the ECSA and (i) CA stability over 8 hours of POP-2.

The careful selection of the monomers resulted in the HOMO–LUMO positions of polymers POP-2 and POP-3 being suitable for overall water splitting (Fig. 3). The bifunctional electrocatalytic performance of the POPs was assessed for both the OER in alkaline medium (1 M KOH) and the HER in acidic medium (0.5 M H_2_SO_4_). Linear sweep voltammetry (LSV) profiles under Ar-saturated conditions at a scan rate of 20 mV s^−1^ (Fig. 4) revealed that the Ni-based POP (POP-2) exhibited a significantly lower OER onset potential (*E*_0_) of 1.45 V *vs.* RHE, outperforming both the metal-free POP-1 (onset of *ca.* 1.75 V) and the Cu-based POP-3 (onset of *ca.* 1.56 V) underlining the role of the metal center. Moreover, the Tafel slope of POP-2 was evaluated to be 167 mV dec^−1^, which is notably smaller than those of POP-1 (294 mV dec^−1^) and POP-3 (242 mV dec^−1^), indicating more favorable kinetics for the OER.^71,72^

The enhanced OER activity of POP-2 can be attributed to the unique electronic and redox properties of the nickel center. Unlike copper, which typically exhibits limited redox activity in the same potential window of the OER,^72^ nickel can reversibly switch between Ni^2+^ and Ni^3+^ (or even Ni^4+^) states during electrocatalysis, which is essential for efficient electron transfer and the formation of O–O bonds in the rate-determining step of the OER. Under alkaline conditions, the surface of nickel-based catalysts often forms Ni(OH)_2_/NiOOH phases, known to be highly active intermediates that facilitate oxygen evolution through a lattice oxygen mechanism. These redox transformations are not only reversible but also fast, contributing to the observed lower overpotential and reduced Tafel slope.^70–72^

POP-2 also demonstrated superior performance for the HER, showing a lower onset potential (*ca.* 159 mV) and a smaller Tafel slope (*ca.* 82 mV dec^−1^) compared to POP-1 (onset of *ca.* ∼176 mV, Tafel slope of *ca.* 89 mV dec^−1^) and POP-3 (onset of *ca.* 215 mV, Tafel slope of *ca.* 248 mV dec^−1^). This remarkable enhancement for hydrogen evolution can also be explained by the strong hydrogen adsorption characteristics of nickel, which offers an optimal binding strength with protons-neither too strong to prevent H_2_ desorption nor too weak to hinder proton adsorption. Furthermore, the integration of Ni into the conjugated carbon framework of POP-2 promotes efficient charge delocalization and electronic communication between the metal center and the conductive matrix, thereby reducing the charge transfer resistance and enabling faster interfacial electron transport. Moreover, the reduction response at *ca.* −0.20 V in POP-1, *ca.* −0.23 V in POP-2 and *ca.* −0.25 V in POP-3 corresponds to the NDI linker unit.^73^ The higher reduction value in POP-2 and POP-3 compared to POP-1 relates to the higher LUMO levels for the POP chelating metal ions.

Electrochemical impedance spectroscopy (EIS) supported these findings. Nyquist plots (Fig. 4) showed that POP-2 and POP-3 exhibited smaller semicircle diameters than POP-1, with POP-2 presenting the lowest charge transfer resistance (*R*_ct_) among the three reported POPs, indicating improved electrical conductivity and rapid charge mobility. The superior performance of POP-2 compared to POP-3 highlights not only the importance of incorporating a metal center, but also the specific advantage of nickel over copper in catalyzing both the OER and HER under the studied conditions.^74^ The atomically dispersed Ni-porphyrin centers (single-site) embedded in the conjugated polyimide framework provide well-defined active sites and facilitate charge delocalization—consistent with the lower *R*_ct_ and higher capacitive signature of POP-2. Long-term stability tests further demonstrated the robustness of POP-2 during continuous electrolysis. In the OER, POP-2 retained 93.7% of its initial current density after 8 h (Fig. 4i), while in the HER, a stable current density of −0.4 mA cm^−2^ was maintained for 8 hours without noticeable performance degradation. Raman spectroscopy and XPS analysis conducted after the stability test revealed no sign of new peak(s) and/or demetallation (Fig. S15), indicating structural integrity.^75^

The Mott–Schottky plot presented in Fig. 4g provides valuable insights into the electronic properties of the porphyrin-based POPs and the impact of metal incorporation (Ni and Cu) on their band structure. The plot reveals the flat-band potential of 0.48 V for POP-1 (metal-free POP), 0.50 V for POP-2 (Ni), and 0.44 V for POP-3 (Cu). The negative slopes observed for all samples confirm their p-type semiconductor behavior, where holes are the majority charge carriers.

The incorporation of Ni induces a slight upward shift in the flat-band potential compared to the metal-free POP (POP-1), consistent with a modulation of the valence band position due to metal coordination. Moreover, the flatter slope observed for POP-2 suggests a higher hole (acceptor) density, which improves electrical conductivity and facilitates a more efficient charge transport. These features, combined with the band alignment obtained from XPS, explain the superior performance of POP-2 for both the OER and HER. The valence band edge of POP-2 is positioned appropriately below the OER level, offering sufficient oxidative driving force, while its conduction band minimum is also favorably placed above the HER level.

In contrast, POP-3, with a slightly lower flat-band potential (0.44 V), shows a valence band position too close to the OER potential, which limits its oxidative efficiency. Although its conduction band minimum lies deeper (–1.0 V *vs.* RHE), providing a favorable potential for HER thermodynamics, the overall catalytic performance of POP-3 remains inferior to that of POP-2. This is likely due to its lower hole density and less efficient charge-transport properties.^76^

To further quantify the density of the active sites, the electrochemically active surface areas (ECSAs) were estimated *via* the electrochemical double-layer capacitance (*C*_dl_). POP-2 showed the highest *C*_dl_ value (19.8 mF g^−1^), exceeding those of POP-3 (15.3 mF g^−1^) and POP-1 (13.9 mF g^−1^), as depicted in Fig. 4h and Fig. S17. This higher *C*_dl_ value reflects a larger number of exposed active sites accessible for the catalytic reaction, which is consistent with the enhanced current densities observed in both the OER and HER for the Ni-based POP (POP-2). Using *C*_s_ = 40 μF cm^−2^, POP-2 shows the largest ECSA of 0.495 cm^2^ (area-normalized) and 495 cm^2^ g^−1^ (mass-normalized), consistent with its higher activity. The turnover frequency (TOF) is calculated at fixed overpotentials using TOF = *IA*/*nFN*_active_ (*n* = 2 HER, *n* = 4 OER) and the conservative site count (*N*_active_) was taken as the total moles of metal per electrode (1.0 mg loading; EDX metal wt%: Ni 39.31%, Cu 19.15%). At *η* = 0.10 V (HER) and *η* = 0.30 V (OER), POP-2 (Ni) reaches TOF of 1.80 × 10^−5^ s^−1^ and 2.61 × 10^−5^ s^−1^, respectively, while POP-3 (Cu) gives TOF of 1.06 × 10^−5^ s^−1^ and 2.59 × 10^−5^ s^−1^.

Finally, a control experiment using monomers TAPP, NiTAPP, and CuTAPP was performed under identical conditions to underline the effect of polymerization, displaying markedly lower electrocatalytic activity than those of the POPs (Fig. S16). Gratifyingly, in acidic HER, NiTAPP did not yield a stable film and dissolved during polarization, highlighting the stabilizing role of the polyimide framework for Ni sites. Overall, the control experiments, TOF analysis, and supporting Mott–Schottky and XPS band alignment data confirm that polymerization not only stabilizes uniformly distributed single-site metal centers but also optimizes the electronic structure and charge carrier density. These combined effects account for the superior bifunctional electrocatalytic performance, particularly in Ni-containing POP-2.

## Conclusions

We have reported successful synthesis of a series of porphyrin-based donor–acceptor porous organic polymers (POPs) through a straightforward polyimide condensation route using tetra(aminophenyl)porphyrins (TAPP, NiTAPP, and CuTAPP) and naphthalenediimide (NTCDA). The resulting frameworks POP-1 (metal-free), POP-2 (Ni(ii)-containing), and POP-3 (Cu(ii)-containing) exhibited well-defined imide linkages, structural integrity, and microcrystalline features as confirmed by FTIR, XPS, PXRD, and solid-state NMR. Optoelectronic studies revealed red-shifted absorption bands and significant band gap reductions relative to their monomeric precursors, highlighting the impact of donor–acceptor engineering on electronic structure modulation. Among the three POP materials, POP-2 demonstrated the most promising bifunctional electrocatalytic activity for both the OER and HER, with lower onset potentials, smaller Tafel slopes, enhanced charge transfer characteristics, and superior electrochemical stability emphasising the critical role of the metal centre, with nickel offering distinct advantages for water-splitting applications. These improvements are attributed to the optimal frontier molecular orbital alignment, higher carrier density, and reversible redox behaviour of the Ni(ii) centre, which facilitate efficient electron transport and catalytic turnover. This study underscores the potential of rationally designed porphyrin-NDI-based POPs with redox-active transition metals, as robust and efficient bifunctional electrocatalysts for sustainable water splitting. The findings open avenues for further exploration of polyimide-linked conjugated frameworks in energy conversion and storage technologies.

## Experimental

### Materials

5,10,15,20-Tetrakis(4-aminophenyl)porphyrin (TAPP), Ni(ii)5,10,15,20-tetrakis(4-aminophenyl)porphyrin (NiTAPP) and Cu(ii)5,10,15,20-tetrakis(4-aminophenyl)porphyrin (CuTAPP) were purchased from Porphychem. 1,4,5,8-Naphthalenetetracarboxylic dianhydride (NTCDA) and anhydrous DMF were purchased from Merck. All the chemicals were used as received without further purification.

### Synthesis of POPs 1–3

5,10,15,20-Tetrakis(4-aminophenyl)porphyrin (TAPP) (50 mg) and 1,4,5,8-naphthalenetetracarboxylic dianhydride (NTCDA) (48 mg) were separately dissolved in anhydrous DMF 4 mL each. Both the solutions were added into a reaction vial (15 mL capacity) and heated at 140 °C for 48 h under dark conditions to afford dark precipitates of POP-1 (yield of 78.0%). The precipitates were filtered off and washed with methanol (10 ml) and acetone (10 ml) followed by frying under vacuum. Similar reaction protocols were adopted to prepare and isolate POP-2 (yield of 82.0%) and POP-3 (yield of 75.0%) using Ni(ii)5,10,15,20-tetrakis(4-aminophenyl)porphyrin (NiTAPP) and Cu(ii)5,10,15,20-tetrakis(4-aminophenyl)porphyrin (CuTAPP), respectively.

### Characterization

The absorbance (*A*) was recorded for the suspensions made after sonication of the monomers and synthesized POPs for 5 minutes in ethanol and was calculated as: *A* = −log(*T*). From the absorbance spectra, the direct optical band gap of the fused-metalloporphyrins was estimated through the Tauc plot as: (*αhv*)^1/*n*^ = *A*(*hv* − *E*_g_), where *α* is the absorbance coefficient, *n* = 1/2 for direct transitions, *h* is Planck's constant and *ν* is the wavelength number.

Scanning electron microscopy (SEM) images were recorded using a FEI Quanta 200F. Transmission electron microscopy (TEM) investigations were carried out using a JEOL – F200 Cold FEG TEM/STEM operating at 200 kV. X-ray photoelectron spectroscopy (XPS) measurements were performed with a Kratos Axis Ultra DLD instrument using a monochromatic Al K_α_ X-ray source of energy 1486.6 eV, at 105 W power. Charge calibration was accomplished by fixing the binding energy of carbon (C 1s) to 285.0 eV.

The adsorption isotherm for N_2_ was measured using a Quantachrome autosorp iQ gas sorption system. Before analysis, samples were degassed at 150 °C for 2 hours and the Brunauer–Emmett–Teller (BET) method was utilized to calculate the specific surface areas. Structural simulation for all the POPs was performed using the Materials Studio (2018) software package.^77^ Firstly, the smallest repeating unit of the POPs was placed in a sql topology crystal cell and geometric optimizations were performed using Universal force field (UFF)-based Lennard–Jones dispersion corrections.^78,79^ Both eclipse AA and staggered AB stacks were considered for all the POPs by creating the corresponding unit cells and their geometry were optimized.

#### Electrochemical characterizations

The POP catalysts were deposited on fluorine-doped tin oxide (FTO) coated glass using a mixture of synthesised POPs (1 mg), Nafion (20 μL) and isopropyl alcohol (20 μL) sonicated for 5 minutes and drop cast on the FTO-coated glass substrates. Linear sweep voltammetry (LSV), cyclic voltammetry (CV) and chronoamperometry measurements were performed with an Autolab PGSTAT302 potentiostat/galvanostat, in a three-electrode configuration cell. The cell consisted of Pt wire as a counter electrode, an Ag/AgCl (3 M KCl) electrode as a reference electrode, and POP coatings on FTO-coated glass as a working electrode. For the OER, 1 M KOH solution at pH 13.1 and for the HER, 0.5 M H_2_SO_4_ solution was used as an electrolyte. Electrodes from TAPP, NiTAPP, and CuTAPP for control experiments were prepared with the same ink formulation and mass loading used for POP films (1 mg catalyst, 20 μL Nafion, 20 μL IPA; drop-cast on 1.0 cm^2^ FTO) and measured in 1 M KOH (OER) and 0.1 M H_2_SO_4_ (HER) using the same three-electrode setup. All the potentials were referred to the reversible hydrogen electrode (RHE) using the Nernst equation: *V*_RHE_ = *V*_Ag/AgCl_ + *V*^0^_Ag/AgCl_ + 0.0591 × pH. Linear sweep voltammetry (LSV) was performed under the relevant electrolyte at a scan rate of 20 mV s^−1^. Electrochemical impedance spectroscopy (EIS) was recorded with a 10 mV AC perturbation over 10 kHz to 10 mHz at the indicated DC bias. The spectra were fitted using ZView software. Mott–Schottky measurements were acquired at 1000 Hz with a 10 mV AC amplitude over a suitable potential range to extract the slope and flat-band potential.

#### Double-layer capacitance (*C*_dl_) and ECSA

Cyclic voltammetry was collected at scan rates of 10–60 mV s^−1^ within a non-faradaic window (0.75–0.95 V *vs.* RHE) to obtain Δ*j*–*ν* plots. *C*_dl_ values were converted to F g^−1^ using a 1.0 mg loading; ECSA was estimated using *C*_s_ = 40 μF cm^−2^. Chronoamperometry (CA) stability tests were performed at fixed potentials relevant to the OER/HER using the same three-electrode configuration.

The geometry optimization and investigation for FMOs of the selected unit in a polymeric structure was carried out in the gas phase at the Becke three-parameter^80^ hybrid exchange functional in concurrence with the Lee–Yang–Parr gradient-corrected correlation function (B3LYP functional)^81^ level of density functional theory (DFT), using the LANL2DZ basis set for POPs 1–3. DFT calculations were performed on all stationary points of the potential energy surface (PES) and studied using the Gaussian 16W suite.^82^

## Author contributions

D. B.: conceptualization, methodology, synthesis, investigation, formal analysis, writing – original draft, writing – review and editing; A. A. N.: investigation, formal analysis, writing – review and editing; S. G.: XRD simulation, writing- review and editing; I. K. P.: writing – review and editing; N. D. B.: formal analysis, funding acquisition, writing – review and editing.

## Conflicts of interest

There are no conflicts to declare.

## Supplementary Material

MA-006-D5MA00957J-s001

## Data Availability

The data supporting this article have been included as part of the SI. See DOI: https://doi.org/10.1039/d5ma00957j.
