# Supplementary material for: Porphyrin-based polyimide 2D porous organic polymers: band engineering for bifunctional electrocatalytic OER and HER
Source: Mater Adv. 2025 Aug 29;6(21):7932–41. doi: 10.1039/d5ma00957j (PMC12461690; doi:10.1039/d5ma00957j)
Supplement: MA-006-D5MA00957J-s001 [file MA-006-D5MA00957J-s001.pdf]

Supplementary Information (SI) for Materials Advances.
This journal is © The Royal Society of Chemistry 2025

Porphyrin-based Polyimide 2D Porous Organic Polymers: Band Engineering for Bifunctional Electrocatalytic OER and HER

Deepak Bansal,*[,a] Amr A. Nada,*[,a] Samrat Ghosh,[b] Indresh Kumar Pandey,[c] Nicolas D. Boscher[a]

[a.] Materials Research and Technology Department, Luxembourg Institute of Science and Technology, 28 Avenue des Hautes-Fourneaux, Esch-Sur-Alzette, Luxembourg.

E-mail: Deepakbans@gmail.com , amr.nada@list.lu

[b] Inorganic and Physical Chemistry Laboratory, Council of Scientific and Industrial Research (CSIR), Central Leather Research Institute (CLRI), Chennai 600020, India.

[c] Department of Chemistry, D.D.U Gorakhpur University, Gorakhpur, Uttar Pradesh

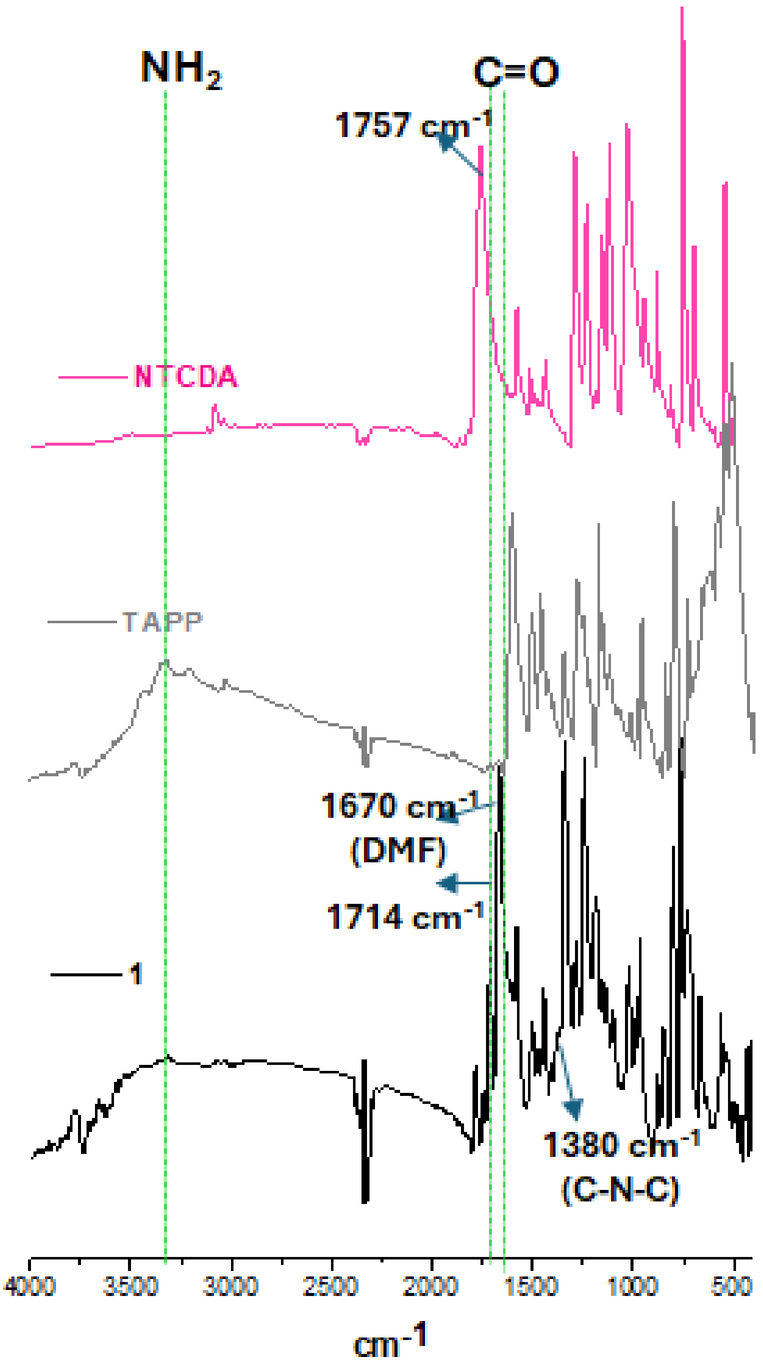

Figure S1. Comparative FTIR spectra NTCDA, TAPP and **POP-1**.

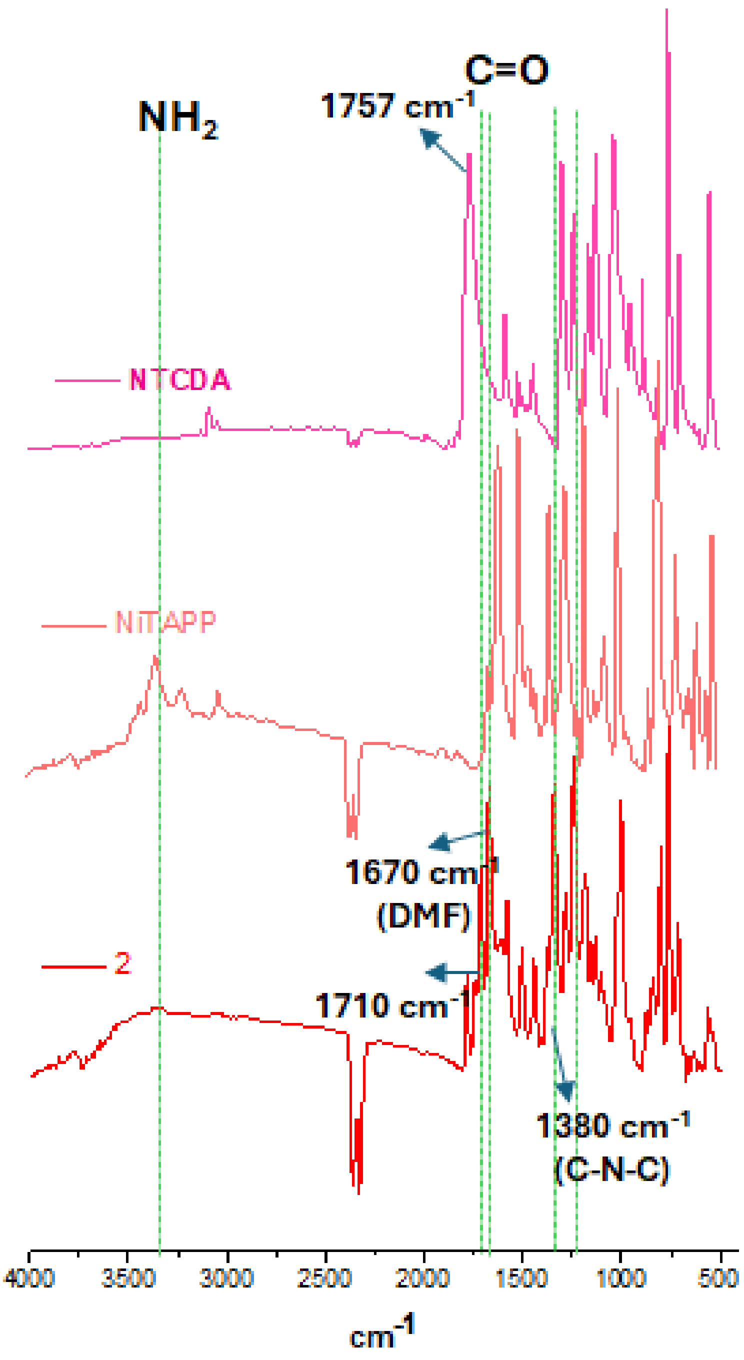

Figure S2. Comparative FTIR spectra NTCDA, NiTAPP and **POP-2**.

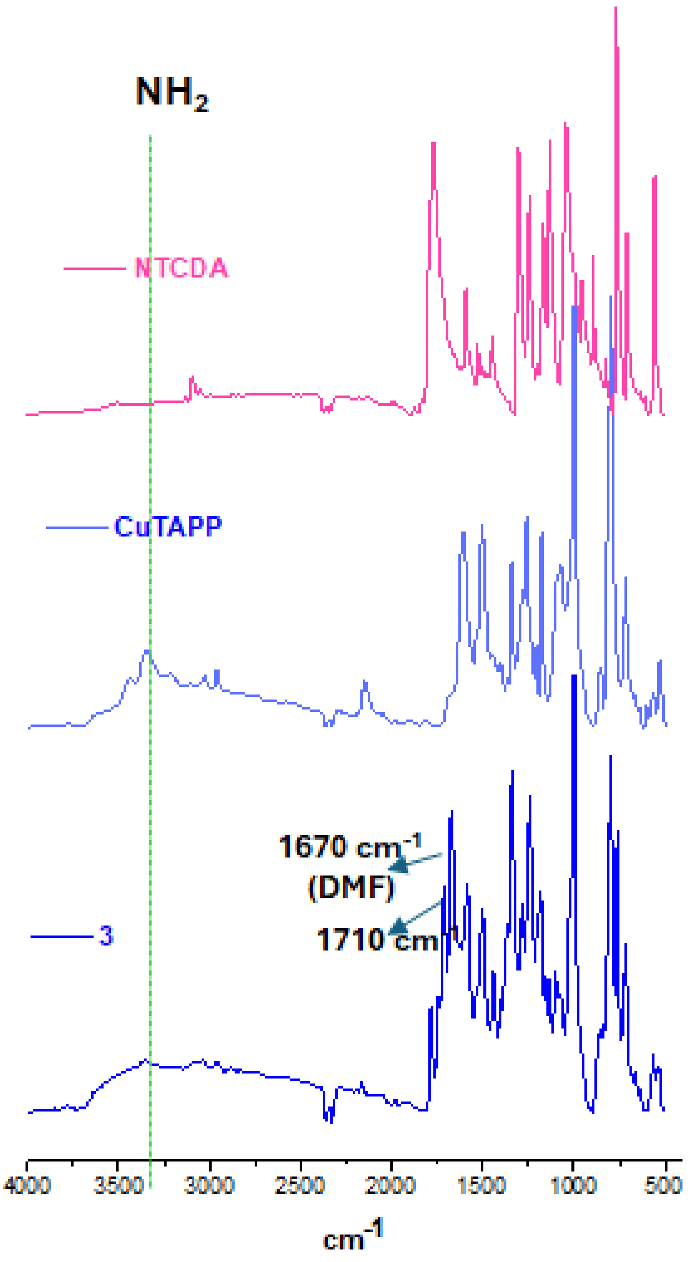

Figure S3. Comparative FTIR spectra NTCDA, CuTAPP and **POP-3**.

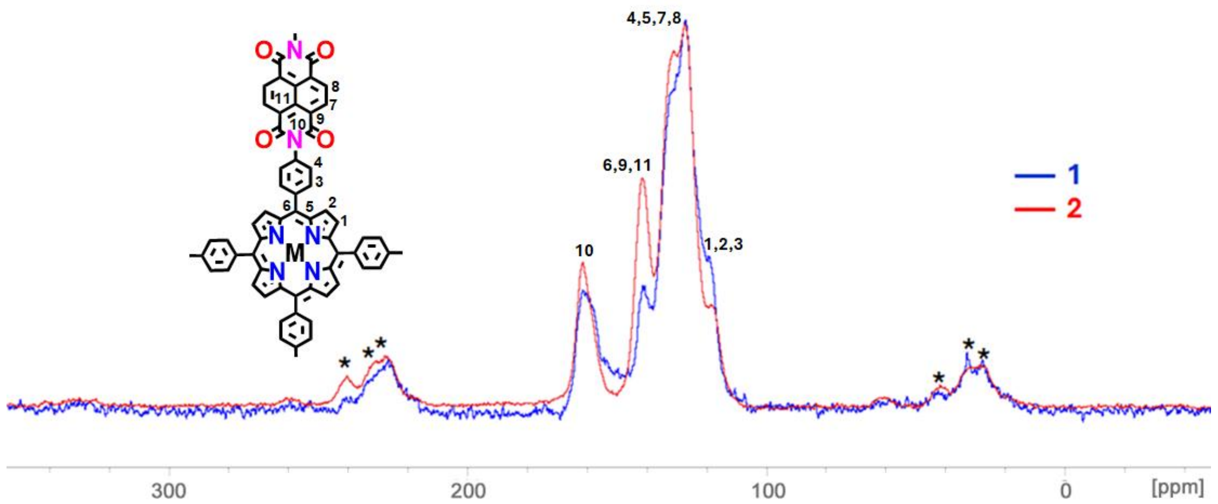

Figure S4. ^{13}C NMR spectra of **POP-1** and **POP-2**.

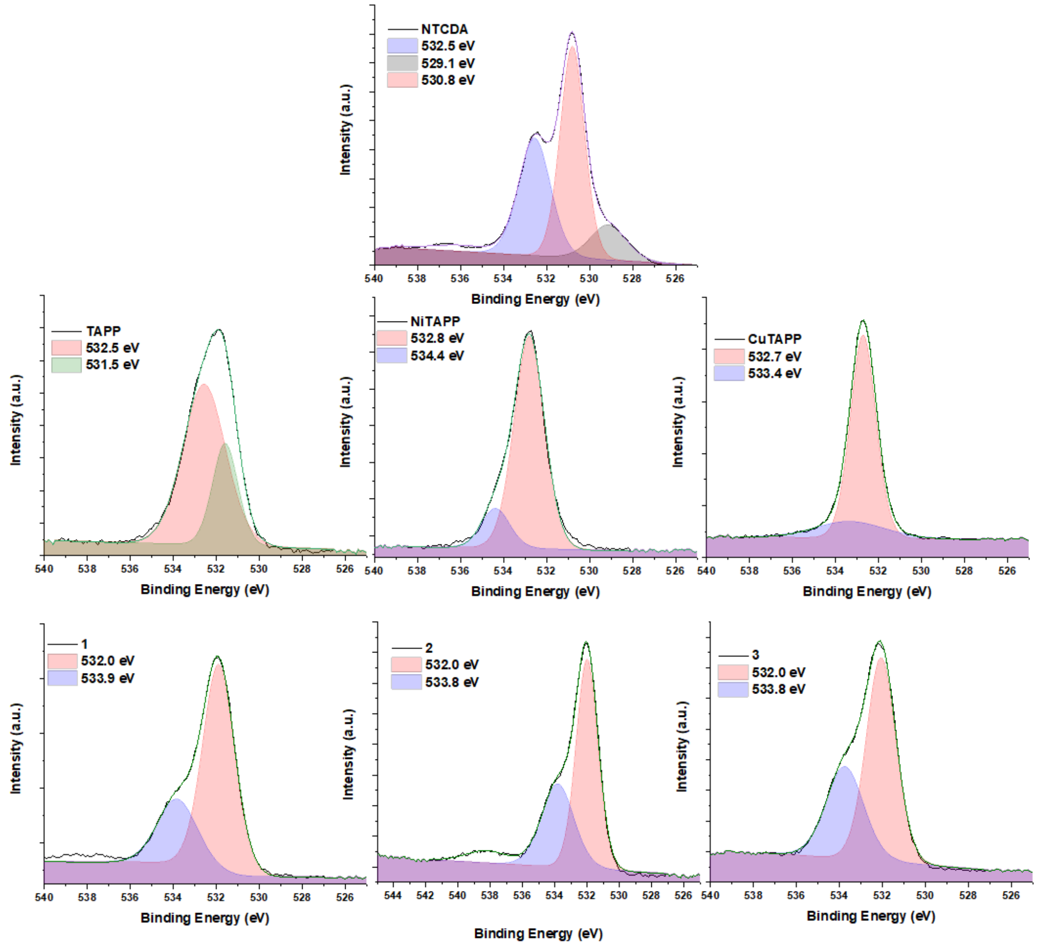

Figure S5. Comparative XPS spectra for the O1s region of NTCDA (top), the aminoporphyrin building blocks (middle) TAPP, NiTAPP and CuTAPP and **POPs 1-3** (bottom).

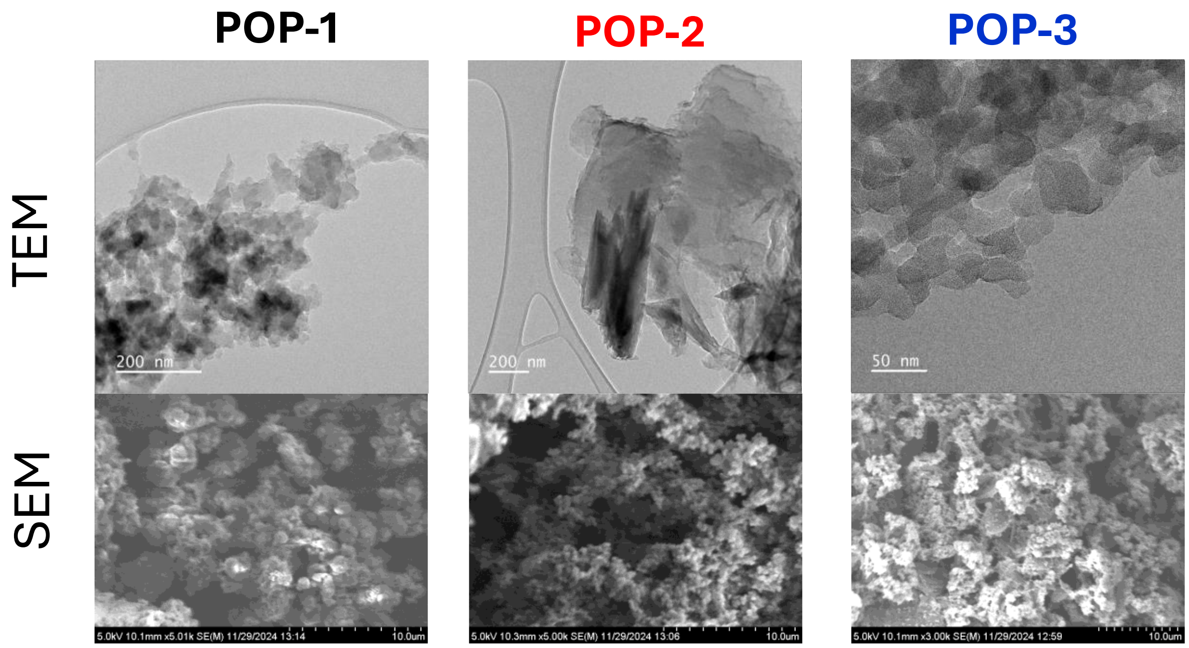

Figure S6. TEM and SEM images of synthesised **POPs 1-3**.

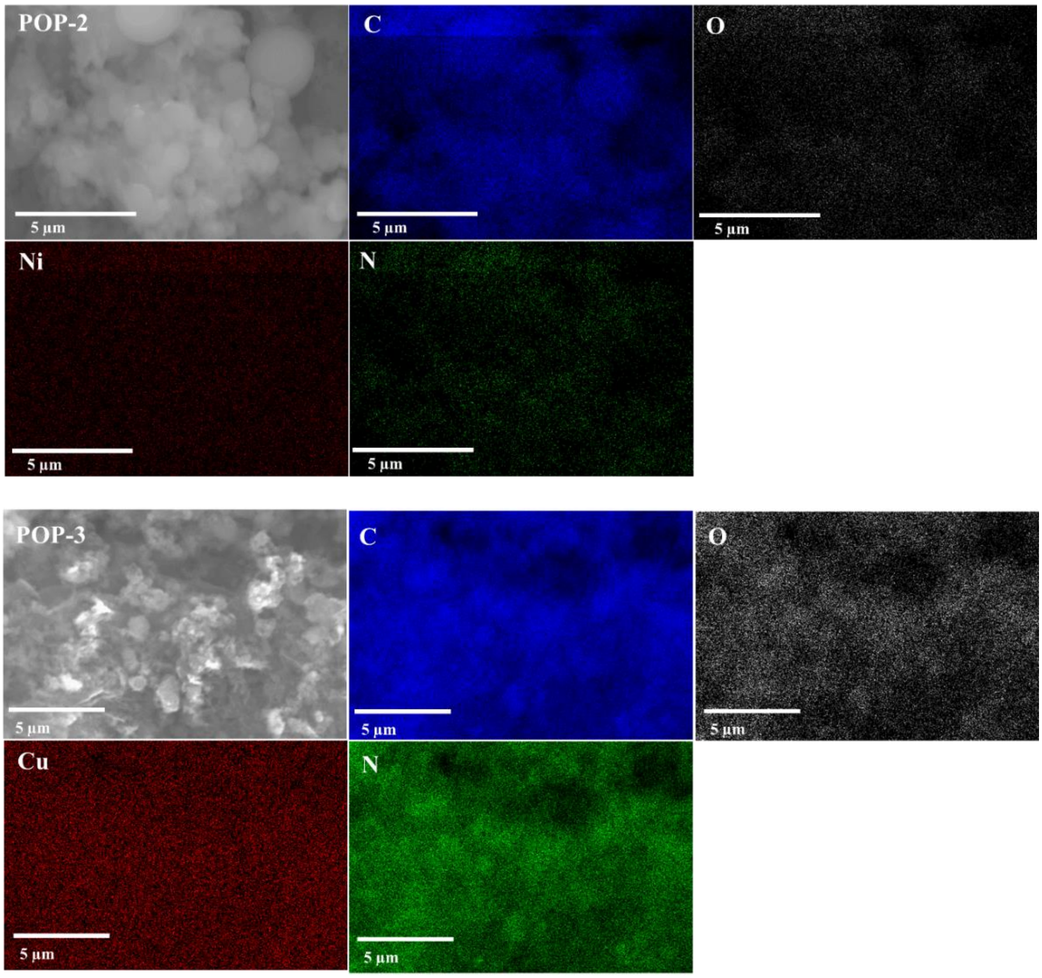

Figure S7. SEM–EDX elemental maps. **POP-2** and **POP-3** maps showing the co-distribution of C, N, O with Ni (POP-2) or Cu (POP-3).

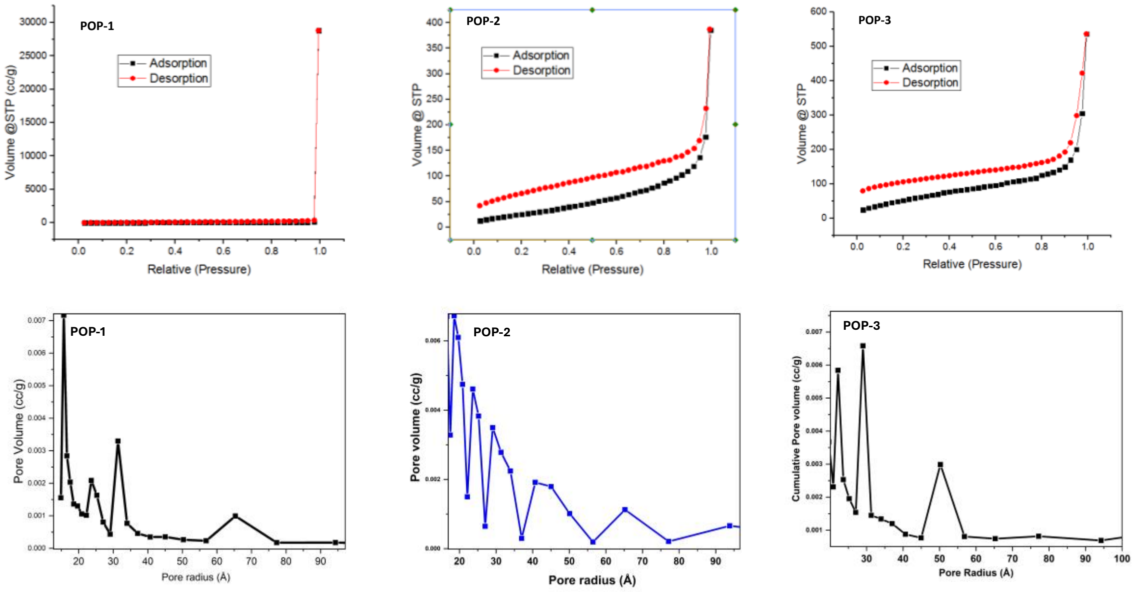

Figure S8. (Top) BET curve for the adsorption and desorption properties and (below) BJH analysis for pore size distribution (below) of **POPs 1-3**.

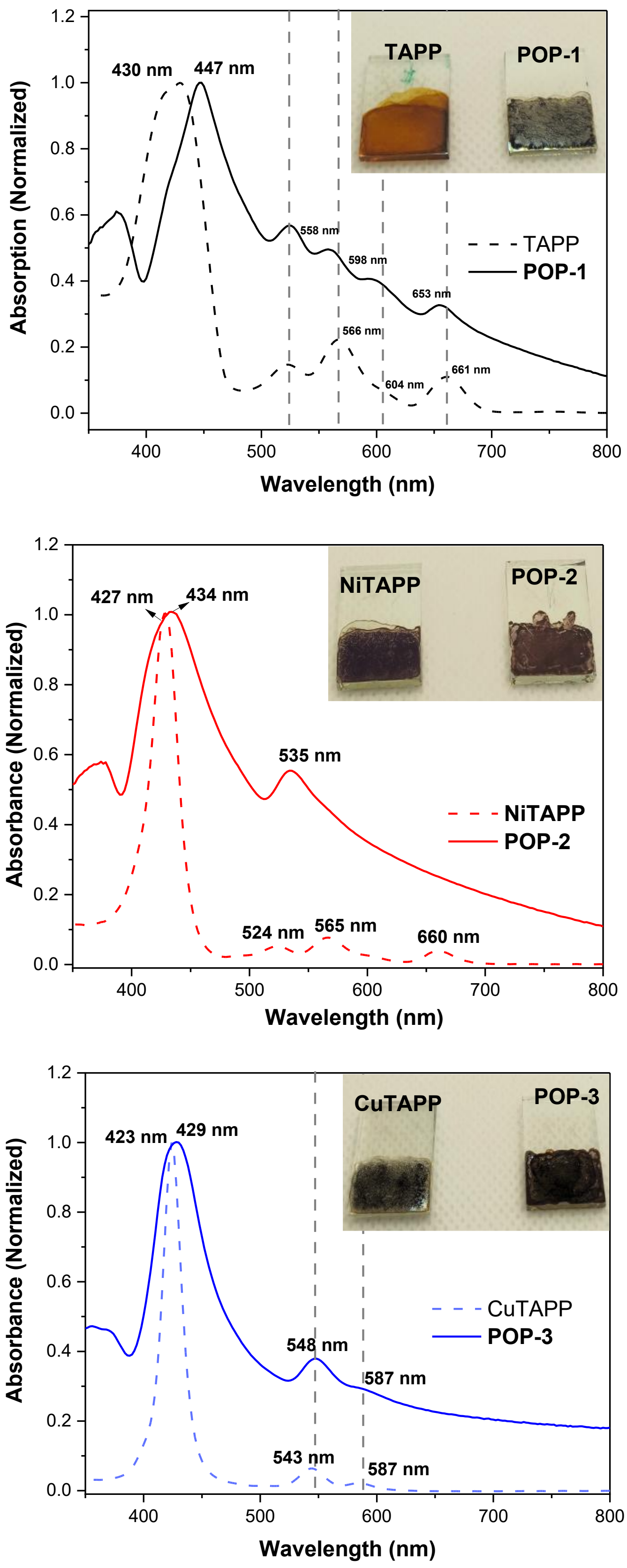

Figure S9. Comparative Diffused reflectance of synthesised **POPs 1-3** and their respective porphyrin building blocks in Ethanol solvent.

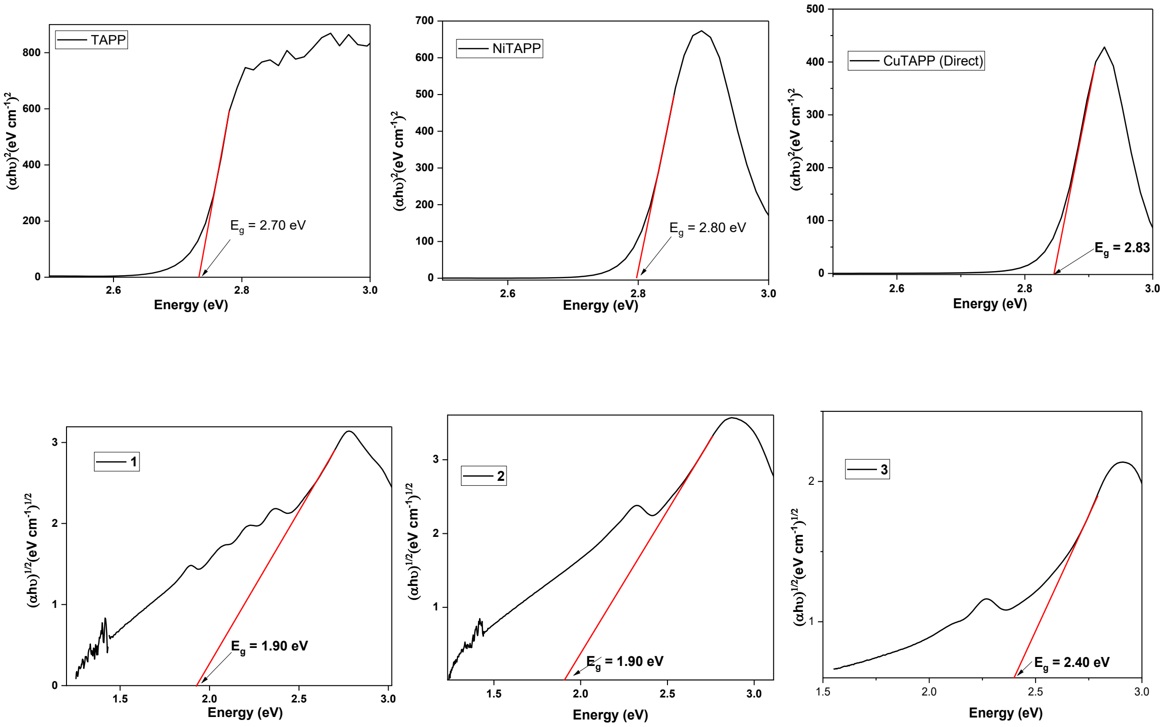

Figure S10. Tauc's plot for TAPP, NiTAPP, CuTAPP and their corresponding **POPs 1-3**.

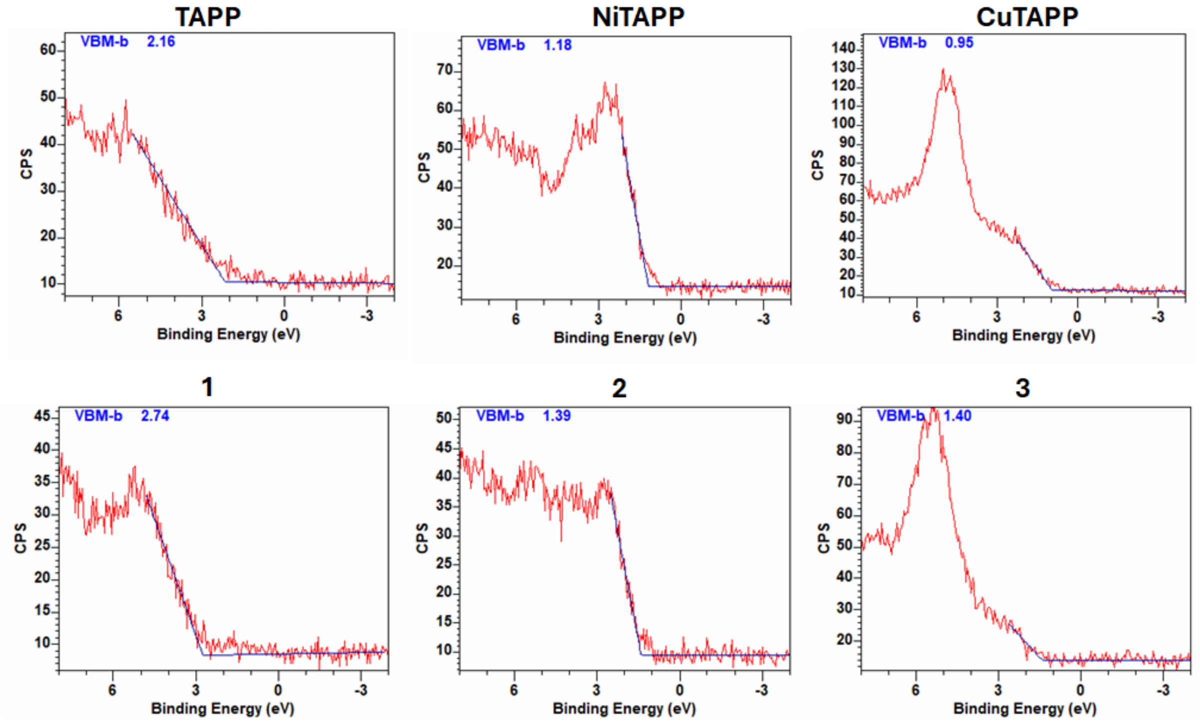

Figure S11. VBM spectra for TAPP, NiTAPP, CuTAPP and their corresponding **POPs 1-3**.

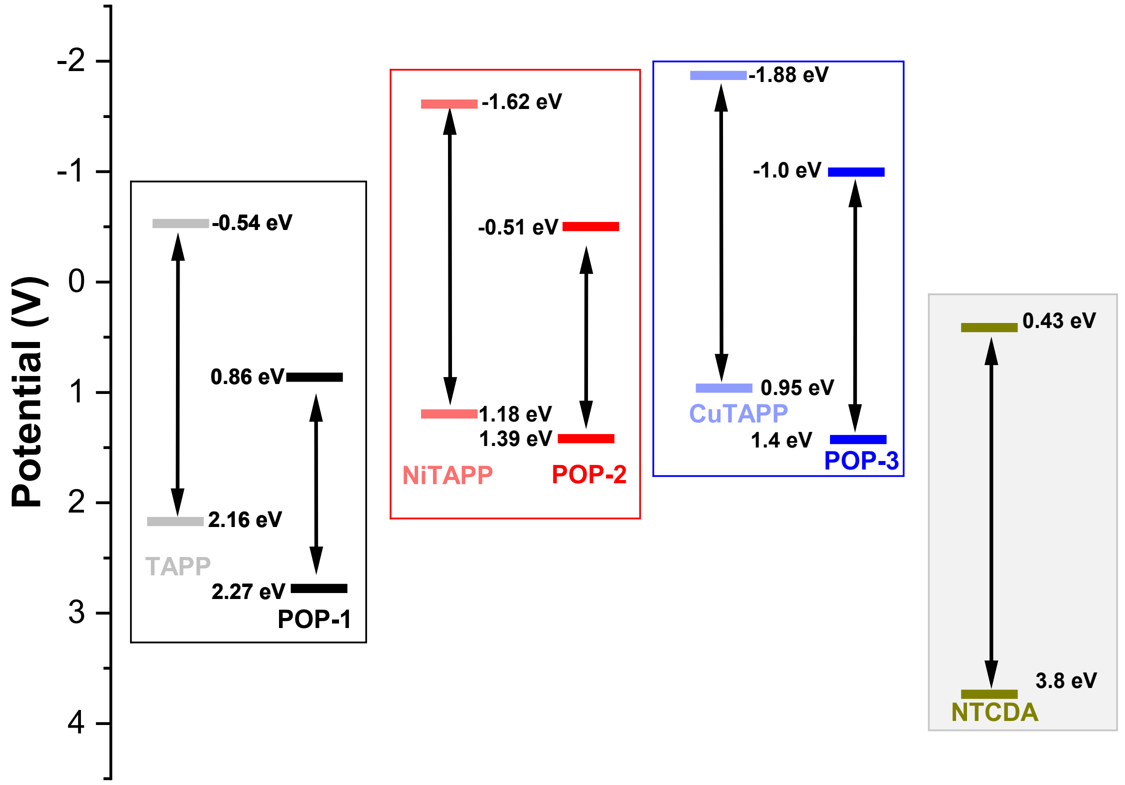

Figure S12. Band diagrams for the monomers NTCDA, TAPP, NiTAPP, CuTAPP and their corresponding **POPs 1-3**.

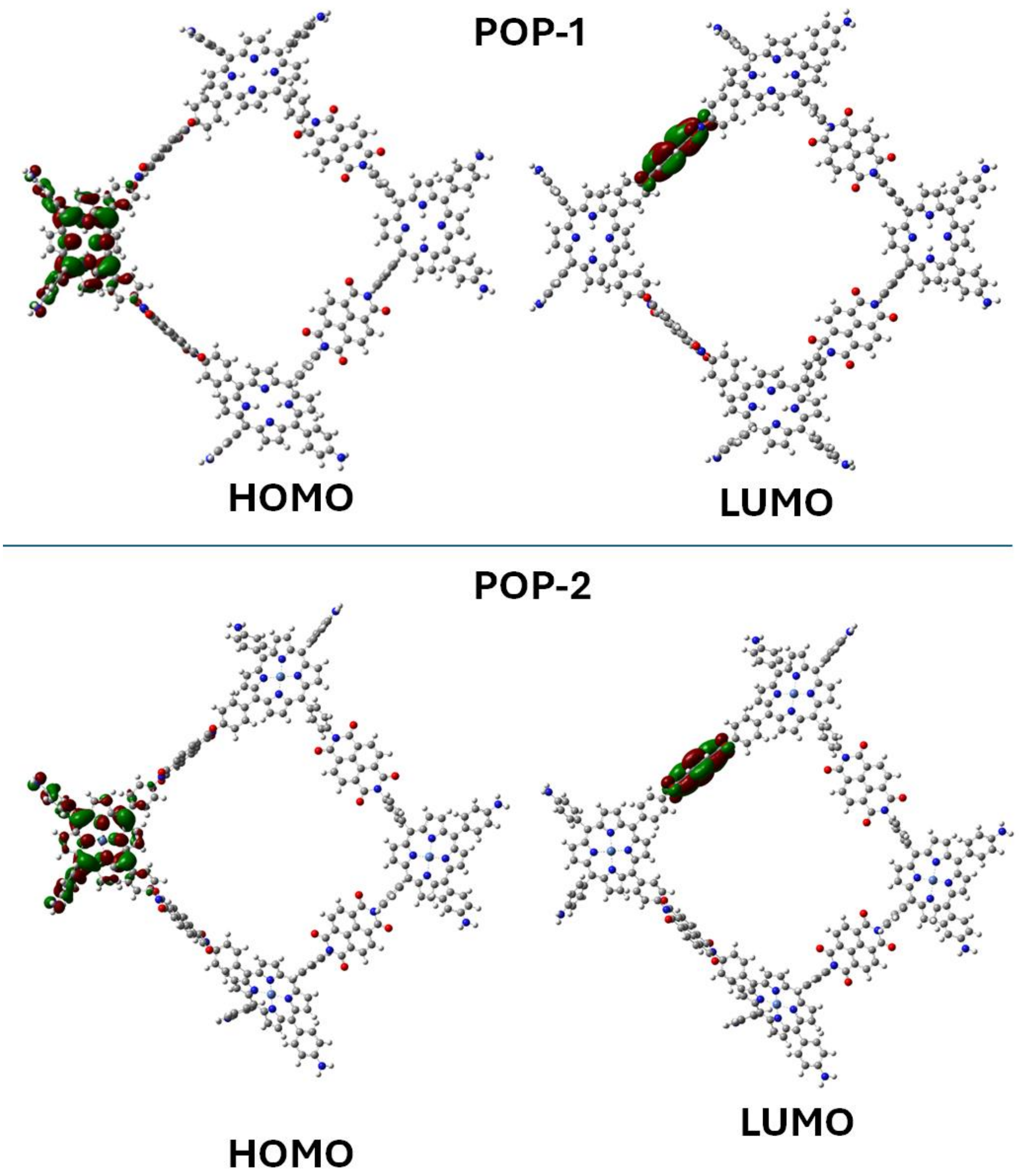

Figure S13. Plot showing HOMO-LUMO distribution in **POP-1** and **POP-2**.

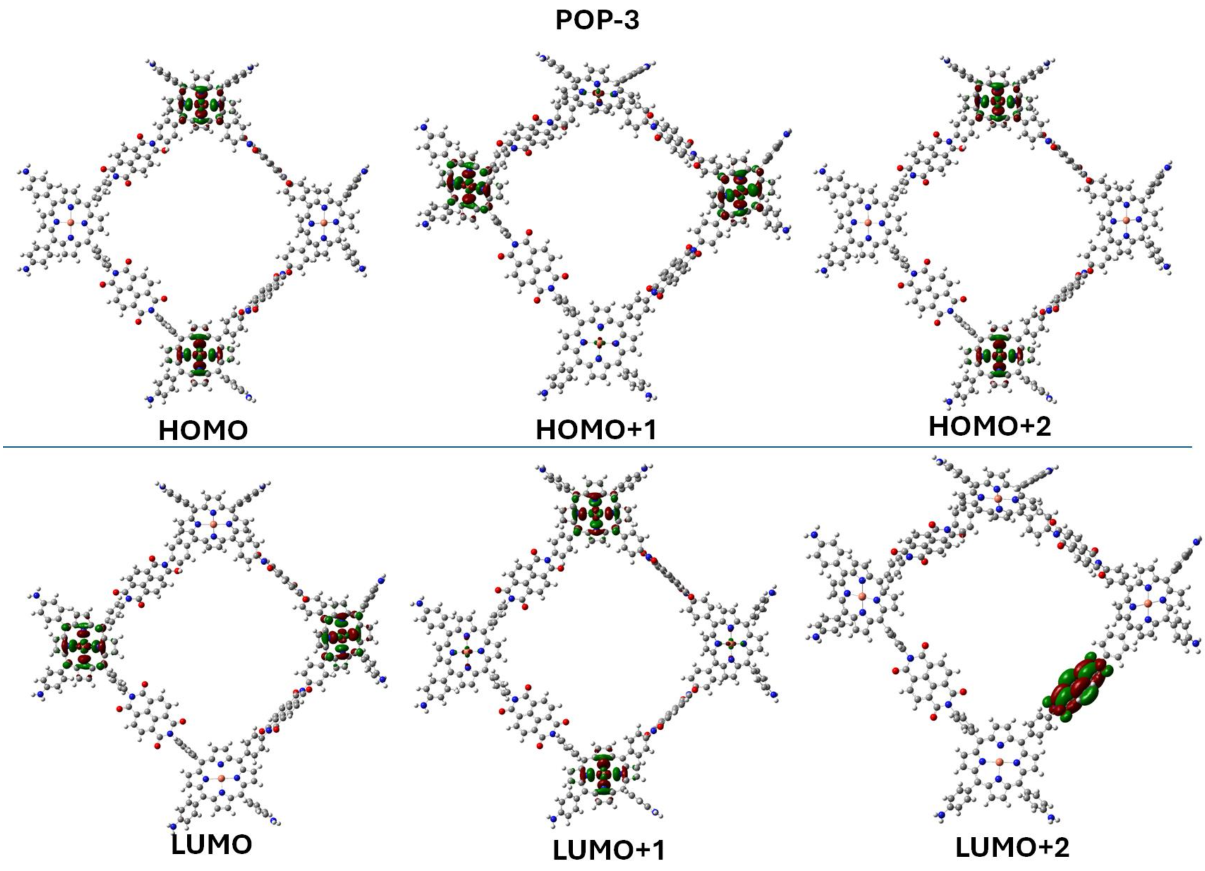

Figure S14. Plot showing HOMO-LUMO distribution in **POP-3**.

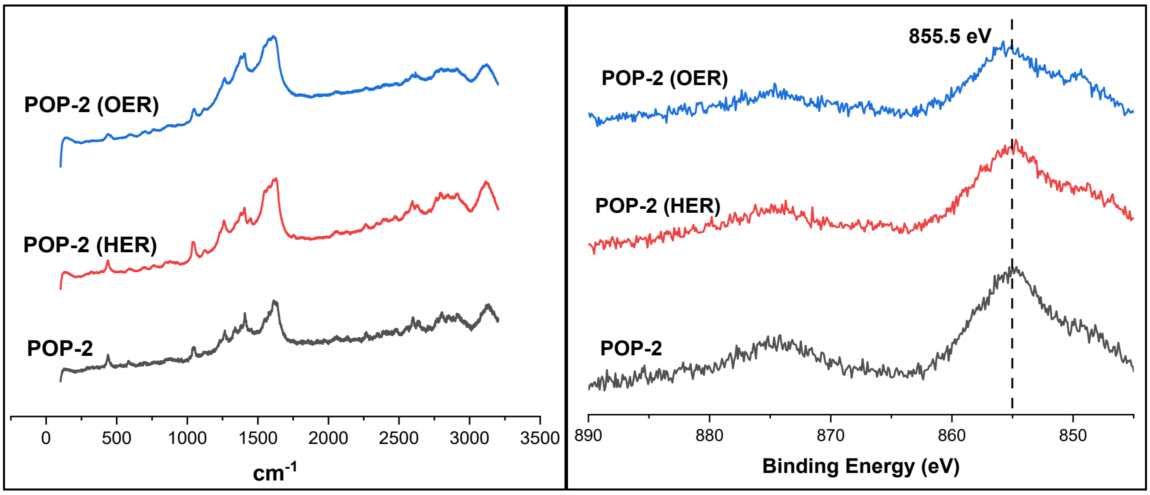

Figure S15. Raman (left) and XPS (right) spectra of **POP-2** before and after HER (**POP-2 HER**) and OER (**POP-2 OER**) stability measurement.

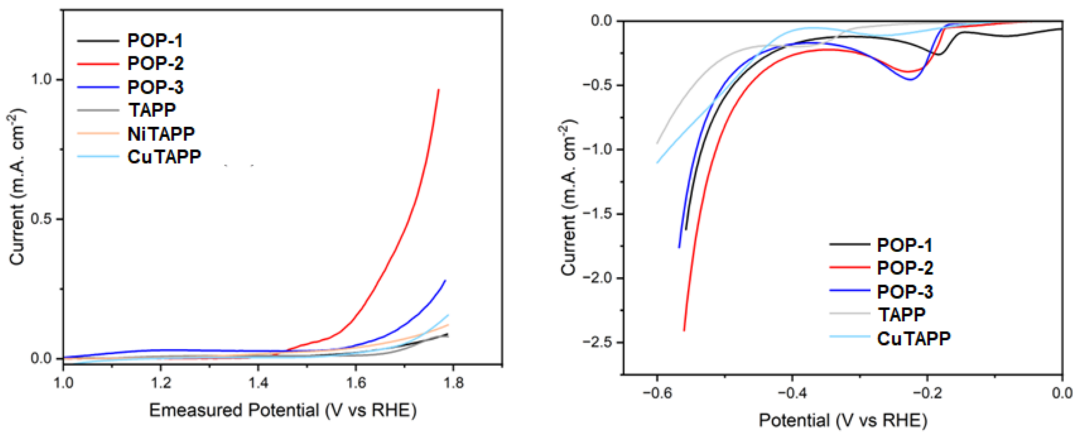

Figure S16. Control electrocatalysis. (a) OER LSVs (1 M KOH) comparing **POPs-1/2/3** with TAPP, NiTAPP, CuTAPP. (b) HER LSVs (0.1 M H_2SO_4) for **POP-1/2/3** and monomers; NiTAPP dissolved under acidic polarization and could not be measured reliably.

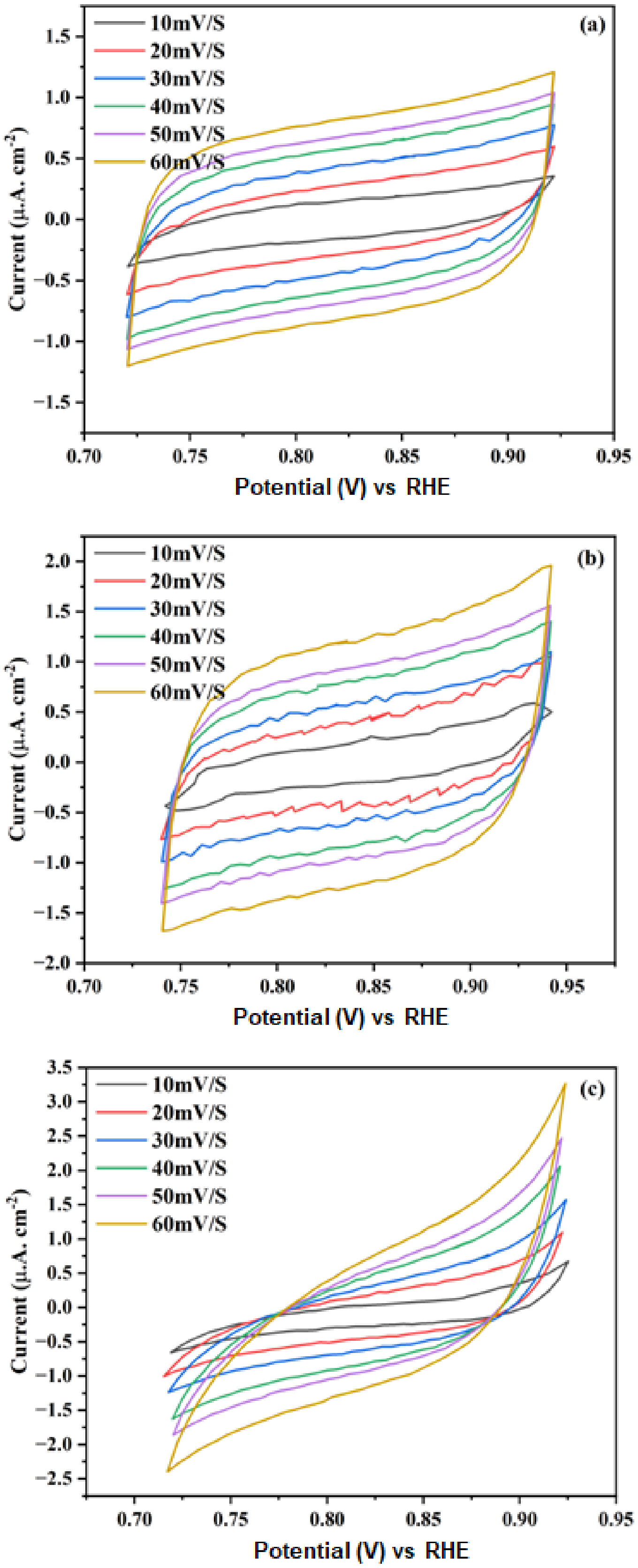

Figure S17. Electrochemical measurement of **POP-1** (a), **POP-2** (b) and **POP-3** (c) at different scan rates.
